# An exploratory study of predisposing genetic factors for DiGeorge/velocardiofacial syndrome

**DOI:** 10.1038/srep40031

**Published:** 2017-01-06

**Authors:** Laia Vergés, Francesca Vidal, Esther Geán, Alexandra Alemany-Schmidt, Maria Oliver-Bonet, Joan Blanco

**Affiliations:** 1 Genetics of Male Fertility Group, Unitat de Biologia Cel·lular (Facultat de Biociències), Universitat Autònoma de Barcelona, 08193-Bellaterra (Cerdanyola del Vallès), Spain; 2 Secció de Genètica Clínica. Hospital Universitari Sant Joan de Déu, 08950-Esplugues de Llobregat, Spain; 3 Laboratori de Genòmica de la Salut, Hospital Universitari Son Espases (HUSE), Palma de Mallorca, Spain

## Abstract

DiGeorge/velocardiofacial syndrome (DGS/VCFS) is a disorder caused by a 22q11.2 deletion mediated by non-allelic homologous recombination (NAHR) between low-copy repeats (LCRs). We have evaluated the role of LCR22 genomic architecture and PRDM9 variants as DGS/VCFS predisposing factors. We applied FISH using fosmid probes on chromatin fibers to analyze the number of tandem repeat blocks in LCR22 in two DGS/VCFS fathers-of-origin with proven 22q11.2 NAHR susceptibility. Results revealed copy number variations (CNVs) of L9 and K3 fosmids in these individuals compared to controls. The total number of L9 and K3 copies was also characterized using droplet digital PCR (ddPCR). Although we were unable to confirm variations, we detected an additional L9 amplicon corresponding to a pseudogene. Moreover, none of the eight DGS/VCFS parents-of-origin was heterozygote for the inv(22)(q11.2) haplotype. PRDM9 sequencing showed equivalent allelic distributions between DGS/VCFS parents-of-origin and controls, although a new PRDM9 allele (L50) was identified in one case. Our results support the hypothesis that LCR22s variations influences 22q11.2 NAHR events, however further studies are needed to confirm this association and clarify the contribution of pseudogenes and rare PDRM9 alleles to NAHR susceptibility.

The human genome is constituted by 5% of segmental duplications (SDs) or low-copy repeats (LCRs). They are defined as near-identical DNA segments (>90% sequence identity), longer than 1 kb and repeated in tandem or interspersed throughout the genome^1^. LCRs contain genes, pseudogenes, repetitive elements and recombination motifs^2^,^3^, which render LCRs unstable and make them prone to misalignments that can lead to non-allelic homologous recombination (NAHR). NAHR events occurring during meiosis result in chromosome reorganizations that can be transmitted to the offspring^4^,^5^,^6^. This process played an important role during primate evolution, which has been deduced from the significant SD expansion in the human genome^7^. NAHR events mediated by LCRs are also responsible for the copy number variations (CNVs) that introduce genetic diversity between individuals^8^. However, some CNVs also originate a group of diseases that have been referred as genomic disorders^9^.

NAHR activity during meiosis can be evaluated in gametes by estimating the rearrangement frequency at a specific locus with polymerase chain reaction (PCR)^10^ or fluorescence *in situ* hybridization (FISH)^11^. Sperm-FISH studies in Prader-Willi syndrome (PWS) and DiGeorge/velocardiofacial syndrome (DGS/VCFS) fathers-of-origin have demonstrated that there are individuals with an increased susceptibility to deletions^12^,^13^. The recurrence risk for PWS and DGS/VCFS in these individuals is considered higher than the general population due to their increased susceptibility to NAHR events during spermatogenesis.

NAHR susceptibility in parents-of-origin has been related to different genetic features. CNVs in the LCRs flanking the critical regions have been described as predisposing factors for Smith-Magenis and Potocki-Lupski syndrome^14^,^15^, Williams-Beuren syndrome^16^ and 16p12.1 microdeletion disease^17^. Inversions of the critical region have been related to the occurrence of Prader-Willi/Angelman syndromes^18^,^19^, Williams-Beuren syndrome^20^,^21^,^22^, Smith-Magenis syndrome^23^, 17q21.31 microdeletions^24^ and t(8;22)^25^, among other genomic disorders^26^. According to these observations, genomic architecture variations in parents-of-origin may confer a higher likelihood of LCR misalignment during meiosis and consequently a higher susceptibility to NAHR events. Moreover, variants of recombination genes have also been associated with altered frequency and stability of meiotic recombination that might also predispose to NAHR^27^,^28^. The PRDM9 protein regulates the pattern of meiotic recombination in the mammal genome by recruiting the double-strand break machinery after recognizing consensus sequences constituted by zinc finger domains^29^,^30^. Nucleotide changes in zinc finger domain shift the binding motif and modify the hot-spot pattern^31^. Therefore, the *PRDM9* genotype determines the likelihood of a NAHR event in a critical region. In this context, some *PRDM9* alleles have been suggested to increase the risk for transmitting Charcot-Marie-Tooth type 1 A, hereditary neuropathy with liability to pressure palsies and Williams-Beuren syndrome, among other genomic disorders^28^,^32^,^33^.

The DiGeorge/velocardiofacial syndrome (DGS/VCFS) (OMIM 188400/OMIM 192430) is the most common deletion syndrome in humans, with an incidence of 1:4000 newborns^34^. The genetic cause is gene haploinsufficiency resulting from a microdeletion of the 22q11.2 region. Roughly 11% of this region is comprised by eight highly homologous LCRs (LCR22-2, LCR22-3a, LCR22-3b, LCR22-4, LCR22-5, LCR22-6, LCR22-7 and LCR22-8)^35^. LCR22s act as substrate for the pathogenic rearrangements that originate DGS/VCFS, as well as a variety of other genomic disorders^36^. Focusing on DGS/VCFS rearrangements, they involve LCR22-2 and LCR22-4 in more than 85% of cases, giving rise to a 3 Mb deletion^37^. NAHR between LCR22-2 and LCR22-3a originates a 1.5 Mb deletion which affects 7% of cases. Distal LCR22s are involved in the remaining DGS/VCFS unusual deletions^38^,^39^,^40^ (Fig. 1A).

Little is known about the variability of the 22q11.2 genomic architecture and its influence in NAHR susceptibility^41^. Some authors have hypothesized about a possible relationship between the frequency of 22q11.2 rearrangements and variations in LCR22s^13^,^42^,^43^. On the other hand, although in other genomic disorders the presence of heterozygote inversions has been associated with an increased frequency of rearrangements, no inv(22)(q11.2) have been described in DGS/VCFS parents^44^,^45^. The influence of the *PRDM9* genotype in the DGS/VCFS transmission risk has also been discussed; nevertheless, an association of *PRDM9* allele and NAHR susceptibility has not been clearly established^32^,^46^.

In this work, we assessed if the risk of DGS/VCFS is influenced by the genomic architecture of LCR22s, the presence of 22q11.2 inversions and the *PRDM9* genotype. First, we analysed CNVs of tandem sequence blocks within LCR22-2 and LCR22-4 and CNVs of paralogous sequences interspersed throughout LCR22s using FISH on extended chromatin fibers (fiber-FISH) and droplet digital PCR (ddPCR), respectively. Second, we developed an interphase FISH assay to genotype inversions between LCR22-2 and LCR22-4. Finally, we analysed the *PRDM9* genotype by sequencing the zinc finger array.

## Results

### LCR22-2 and LCR22-4 tandem sequence blocks

**Figure 1:**
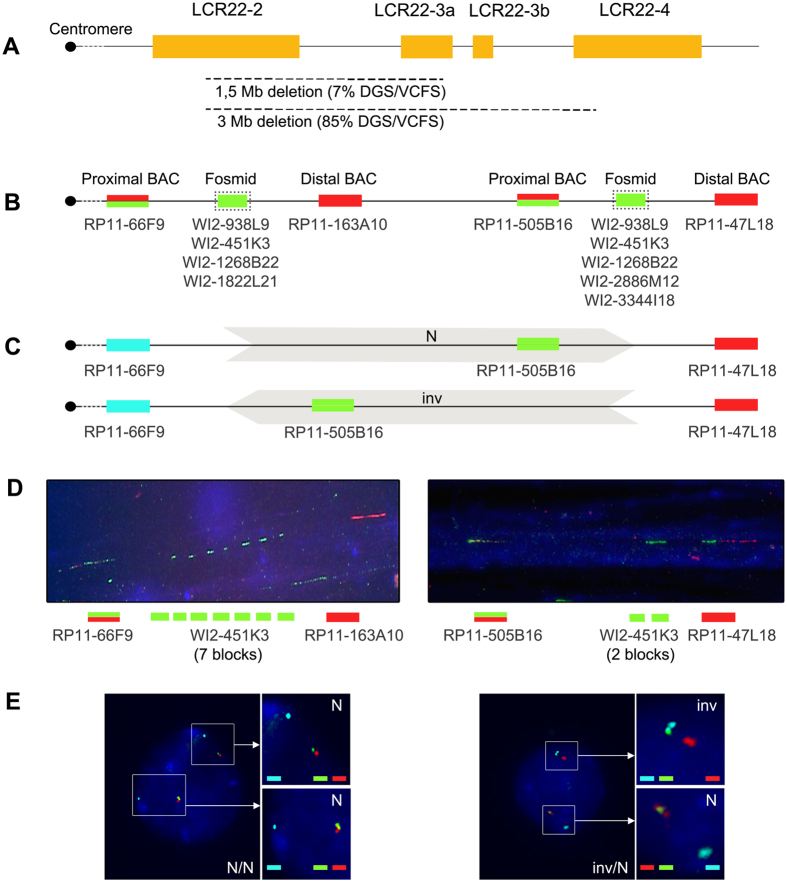
Experimental approach to assess the genomic architecture of DGS/VCFS critical region. (**A**) Schematic representation of the DGS/VCFS critical region. Main deletions mapped are in broken lines. (**B**) BAC and fosmid probe combinations for the study of LCR22-2 and LCR22-4 using fiber-FISH. The three co-hybridized probes are unequivocally labelled to visualize the copy number variations of each fosmid sequence in a single LCR: proximal BAC (red and green), fosmid (green) and distal BAC (red). (**C**) BAC probes to study 3 Mb inversions between LCR22-2 and LCR22-4. Normal haplotypes (N) present clustered RP11-505B16 and RP11-47L18 signals (green and red) and a separated RP11-66F9 signal (blue). 3 Mb inversion haplotypes (inv) present clustered RP11-66F9 and RP11-505B16 signals (blue and green) and a separated RP11-47L18 signal (red). (**D**) Block copy number of WI2-451K3 in LCR22-2 (left) and in LCR22-4 (right) observed by fiber-FISH. (**E**) Normal (N) and inverted (inv) haplotypes ascertained by interphase FISH in lymphocyte nuclei. Example of three normal (N) and one inverted haplotype (inv) in two lymphocyte nuclei.

The LCR22-2 and LCR22-4 genomic architecture was analysed in lymphocyte stretched chromatin fibers using fosmid and single-copy BAC clones (Human genome February 2009 assembly; UCSC Genome Browser; http://genome.ucsc.edu/) (Fig. 1B). Single copy BAC clones were selected to delimit the proximal and distal ends of LCR22-2 (RP11-66F9 (abbr. F9) and RP11-163A10 (abbr. A10)) and LCR22-4 (RP11-505B16 (abbr. B16) and RP11-47L18 (abbr. L18)). The experimental design also required the use of fosmid clones covering the length of LCR22-2 (WI2-938L9 (abbr. L9), WI2-451K3 (abbr. K3), WI2-1268B22 (abbr. B22) and WI2-1822L21 (abbr. L21)) and LCR22-4 (WI2-938L9 (abbr. L9), WI2-451K3 (abbr. K3), WI2-1268B22 (abbr. B22), WI2-2886M12 (abbr. M12) and WI2-3344I18 (abbr. I18)) (Fig. 1B and Supplementary Table S1).

**Figure 2:**
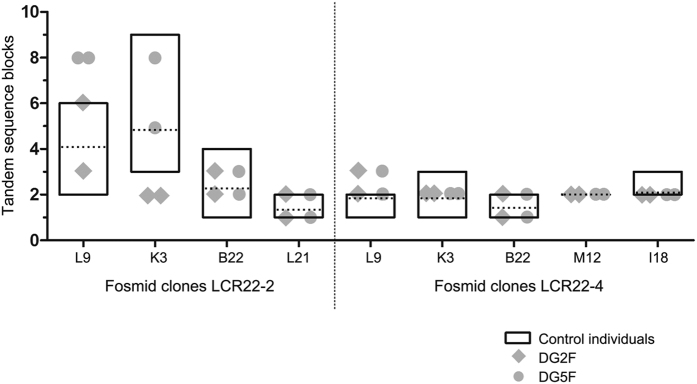
Fiber-FISH analysis of tandem sequence blocks of LCR22-2 and LCR22-4. The floating bar graph represents CNVs of tandem sequence blocks in control individuals. Boxes mark the minimum and maximum values while dashed lines represent the mean. Data from DGS/VCFS fathers-of-origin with increased NAHR susceptibility at 22q11.2 (DG2F and DG5F) are superimposed over the graphic.

Normal ranges of tandem sequence blocks were established by taking the smallest and the largest CNVs observed within six control individuals. For LCR22-2, normal ranges were 2–6 copies for L9, 3–9 copies for K3, 1–4 copies for B22 and 1–2 copies for L21; for LCR22-4 were 1–2 copies for L9, 1–3 copies for K3, 1–2 copies for B22, 2–2 copies for M12 and 2–3 copies for I18 (Table 1; Fig. 2). Two DGS/VCFS fathers-of-origin with a previously proven 22q11.2 NAHR susceptibility were also examined^13^. Both fathers showed out of range numbers of fosmid blocks. DG2F presented two copies of K3 blocks in both LCR22-2 haplotypes rather than the 3–9 copies in the controls, and three copies of L9 blocks in one LCR22-4 haplotype rather than the 1–2 copies in controls. Otherwise, DG5F presented eight copies of L9 blocks in both haplotypes from LCR22-2 rather than the 2–6 copies in the controls, and three copies of L9 blocks in one haplotype from LCR22-4 rather than the 1-2 copies in controls (Table 1; Fig. 2).

### LCR22 paralogous sequences

To validate the fiber-FISH results, the total number of L9 and K3 blocks scattered around LCR22s was analysed by ddPCR using lymphocyte genomic DNA. This was done in those samples previously analysed by fiber-FISH (C1, C2, C3, C4, C5, C6, DG2F and DG5F). The L9 and K3 ddPCR results did not correlate with fiber-FISH (Spearman correlation test, r = 0.156 and 0.386, *P* = 0.703 and 0.360, respectively).

**Figure 3:**
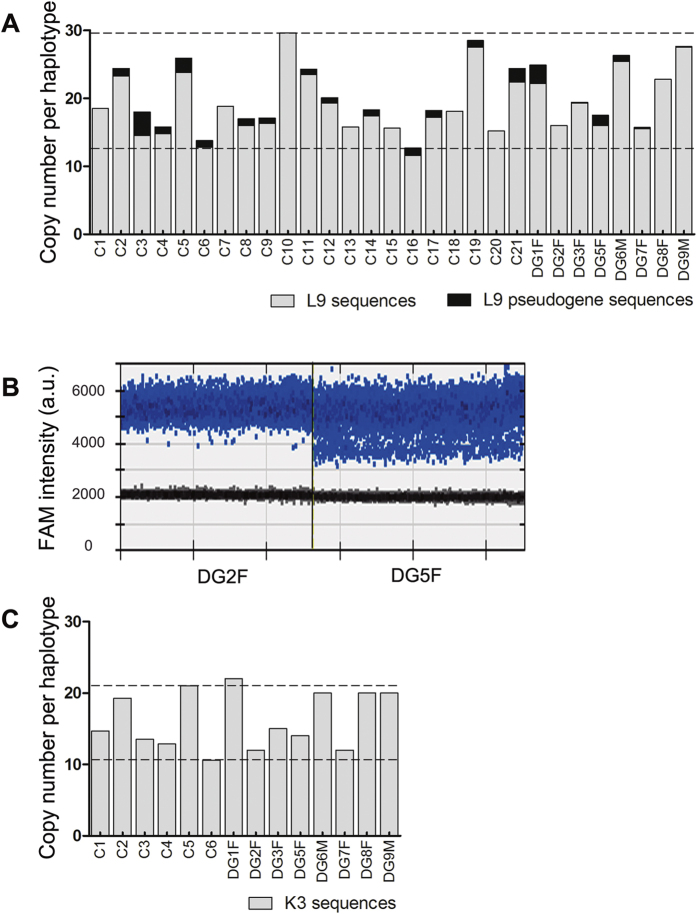
Quantification of copies of LCR22s paralogous sequences by ddPCR in control individuals and DGS/VCFS parents-of-origin. (**A**) Mean L9 sequences (grey bars) and L9 pseudogene sequences (black bars) per haplotype. Dashed lines represent the limits of the normal range observed in controls (C1–C21). (**B**) ddPCR results showing different types of amplicons for L9 fosmid sequence. DG2F shows a single group of positive droplets, while DG5F shows a second group of positive droplets with lower fluorescence amplitude. (**C**) Mean K3 sequences per haplotype. Dashed lines represent the limits of the normal range observed in controls (C1–C6).

Next, we determined the normal range of L9 and K3 copies in 21 and 6 control samples respectively. The L9 range extends from 12.7 to 29.6 (Fig. 3A), while K3 extends from 10.6 to 21 (Fig. 3C). The mean L9 copy number in controls and in the eight DGS/VCFS parents-of-origin was 19.54 and 21.28 respectively, while the mean K3 copy number was 15.33 in controls and 17.46 in DGS/VCFS parents-of-origin. However, L9 and K3 copies failed to reach significant differences between both groups (Mann Whitney test, *P* = 0.420 and 0.477, respectively). At the individual level, only DG1F obtained a K3 copy number above the normal range observed in controls (22 copies) (Fig. 3A).

Interestingly, we detected two types of amplicons for L9 fosmid which were differentiated by the fluorescence amplitude (Fig. 3B). It has been described that droplets with lower fluorescence amplitude are amplicons of different size that may correspond to pseudogene sequences^47^. 66.67% of control individuals (14 of 21) presented two or more copies per genome of the L9 pseudogene, while in DGS/VCFS parents-of-origin this figure reached only 37.50% (3 of 8) (Fig. 3A). However, these differences did not reach statistical significance (Fisher exact test, *P* = 0.218).

### 22q11.2 inversions

22q11.2 inversions were evaluated in five control individuals and eight parents-of-origin. We performed an assay using a combination of three BAC clones including RP11-66F9 (abbr. F9) covering the proximal end of the LCR22-2 and RP11-505B16 (abbr. B16) and RP11-47L18 (abbr. L18) covering the proximal and the distal end of the LCR22-4, respectively (Fig. 1C and Supplementary Table S1).

A total of 1354 informative signal combinations, 512 from control individuals and 842 from DGS/VCFS parents-of-origin, were analysed (Supplementary Table S2). The mean frequency of normal haplotypes was 96.08% in control individuals (range 94.12% to 98.06%) and 96.92% in DGS/VCFS parents-of-origin (range 94.55% to 99.02%). The mean frequency of the 3 Mb inversion haplotype was 1.57% in control individuals (range 0.96% to 2.94%) and 1.56% in DGS/VCFS parents-of-origin (range 0% to 2.91%). We also detected a negligible percentage of informative haplotypes with an unexpected probe association attributed to inherent hybridization errors (Supplementary Table S2).

The frequency of 3 Mb inversions was statistically significant lower than the frequency expected in a heterozygous situation in all the cases (Chi square-test, *P* < 0.05) (Table 2 and Supplementary Table S2). Haplotype frequencies were not different between both populations (Mann Whitney test, *P* > 0.05).

### *PRDM9* genotype

**Figure 4:**
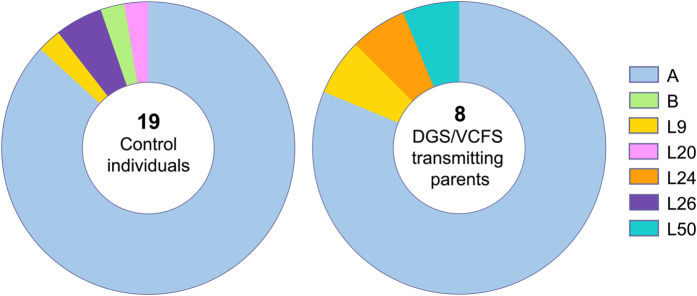
Distribution of *PRDM9* alleles in control individuals and DGS/VCFS parents-of-origin.

Sequencing of the *PRDM9* zinc finger array was performed in 19 control individuals and eight DGS/VCFS parents-of-origin. We observed the common A allele, constituted by 13 zinc finger repeats (ZF), in 86.84% of our control population and in 81.25% of the case population. One new allele showing more than 13 ZF was identified (L50); unfortunately, we were unable to define its ZF combination due to technical limitations (Fig. 4 and Supplementary Table S3).

Control and case allele frequencies did not show differences (Fisher’s exact test, *P* > 0.05). DGS/VCFS fathers showing an increased NAHR susceptibility at 22q11.2 (DG2F and DG5F) presented the A/L50 and A/A genotypes, respectively.

## Discussion

The recurrence risk for transmitting genomic disorders is generally considered not higher than that for the normal population. However, some insights have been provided into the predisposing genetic factors for these diseases. Our exploratory analyses reveal that the LCR22 genomic architecture, through CNVs of the segmental duplication blocks, could be a predisposing factor for transmitting DGS/VCFS.

LCR sequences present a poor precision within databases, which complicates the knowledge of their structural variation. Their repetitive nature constitutes an important impediment for accurate sequencing procedures. Nonetheless, different approaches, such as massively parallel sequencing techniques, PCR or FISH, have been applied to analyse repetitive regions^16^,^48^,^49^,^50^,^51^. Fiber-FISH provides a direct analysis of genome architecture with high resolution, i.e. 10–20 kb^52^. Moreover, the visualization of repeating patterns allows for identifying heterozygote haplotypes and analyzing LCRs individually. For these reasons, fiber-FISH is the preferred method for studies of the genomic structure of complex CNVs^53^. However, it cannot differentiate tandem copies of the regions recognized by the fosmids. Accordingly, we had to consider that, in the visualization of repeated patterns, each signal corresponds to a block of tandem sequences of each fosmid (Fig. 1C). Despite overtaking the identification of tandem sequences and detecting pseudogene sequences, PCR-based methods cannot differentiate heterozygote haplotypes or analyse variations at the LCR level.

CNVs within LCRs have been previously reported in a database of genomic variants, thus demonstrating their polymorphic length^54^. Our ddPCR results reinforce this interpretation since the data show the inter-individual variability of LCR22 repeated sequences. Moreover, the fiber-FISH results show that LCR22-2 inter-individual variability was higher than that of LCR22-4. Focusing on those fosmid clones analysed in both LCRs (L9, K3 and B22), LCR22-2 showed larger ranges of tandem sequence blocks per fosmid, especially from those blocks corresponding to tandem sequences of L9 and K3. On the contrary, LCR22-4 showed less variable numbers for the five fosmids analysed with restricted ranges of copies and minor differences between individuals (Fig. 3, Table 1). These observations suggest that LCR22-2 is longer than LCR22-4. Indeed, LCR22-2 mediates the majority of rearrangements affecting the 22q11.2 region and is the LCR22 with the highest rates of allelic homologous recombination (AHR)^43^. Probably a longer LCR22 might contain more recombination motifs, which what would explain its higher AHR and also the increased likelihood of misalignments and NAHR events. Concordantly, LCR length, homology degree of segmental duplication blocks and the density of recombination motifs have been related to the NAHR frequency of recurrent rearrangements^3^.

In DGS/VCFS fathers-of-origin, we identified abnormal CNVs of the tandem sequence blocks for L9 and K3 fosmids. We previously demonstrated that these individuals showed significant increases of sperm 22q11.2 deletions^13^. In both cases, the deletion in the affected children correspond to the 3 Mb segment between LCR22-2 and LCR22-4^13^. Furthermore, parallel investigations in the same individuals determined the rearrangement frequency at other critical regions with a similar genomic architecture by applying sperm FISH experiments based on the methodology described by Molina *et al*.^11^. These studies demonstrated that NAHR susceptibility was restricted at the DGS/VCFS critical region because they presented normal deletion and duplication sperm rates for the Williams-Beuren syndrome and the Prader-Willi syndrome regions (7q11.23 and 15q11-q13) (Table 2; unpublished data). Altogether, our results support the association between increased rates of rearrangements during spermatogenesis and specific genetic features of the critical region and reject a generalized instability origin for these abnormal deletion rates.

CNVs within LCRs affect the number of different types of sequences such as genes, pseudogenes and recombination motifs. The L9 and K3 sequences (UCSC Genome Browser) encompass partial or complete genes and pseudogenes; L9 covers *USP18* and *AK129567*, whereas K3 covers *AK302545* and *GGT3P*. The density of *PRDM9* A binding motifs (CCNCCNTNNCCNC) is three on L9 and two on K3. It is important to remark that the gene *AK129567* covered by L9 sequence belongs to the common breakpoint region of the 22q11.2 deletion^55^. The most frequent breakpoints at LCR22-2 and LCR22-4 occur in regions including *DGCR6, PRODH* and *AK129567* at the proximal end and *GGT2, HIC2, RIMBP3* and *AK129567* at the distal end^55^. These regions are also covered by the fosmid sequences L21 and M12 which, despite presenting a low variation in our analyses, contain higher sequences of recombination motifs (eight and 14, respectively) (Supplementary Table S4). It has been demonstrated that the *PRDM9* recombination motif density at LCRs has a positive correlation with the NAHR frequency. Specifically, structural variants generated by NAHR present breakpoints enriched with *PRDM9* defined hotspots^3^,^28^,^29^. Hence, the concentration of *PRDM9* recombination motifs may define the location of the rearrangement breakpoints, whereas the misalignment probability could be driven by variations in the degree of homology between paralogous sequences. L9 and K3 CNVs might produce variations in the degree of homology between LCR22-2 and LCR22-4, leading to non-allelic alignments of the susceptible breakpoint regions during meiotic prophase I that would be solved in NAHR events. Instead, the DSB formation to start a NAHR event might be driven by adjacent sequences with a high recombination motif density, such as L21 or M12 sequences.

Different authors have reported a correlation between the alignment length and the sequence identity between paired LCRs and NAHR frequency^3^,^56^, thus supporting the idea that CNVs at LCRs might change NAHR susceptibility. Moreover, according to the ectopic synapsis model^15^, variations in the length of the LCRs might originate ectopic presynaptic contacts between near homologous segments that would act as a precursor to NAHR.

Regarding the total number of paralogous LCR22s sequences, the ample spectrum of CNVs detected endorse the polymorphic variation of these sequences and the uncertainty of the human reference assemblies in LCR regions. Comparing the control and DGS/VCFS parent-of-origin populations, we did not find any relationship between the CNVs of paralogous sequences and the risk for transmitting DGS/VCFS. At the individual level, we observed one DGS/VCFS father-of-origin with a CNV of K3 sequences out of the control range. However, considering that this father did not show NAHR susceptibility in previous studies^13^ and that, due to the technical characteristics of ddPCR, we could not attribute the higher copy number to a specific LCR22, we believe that this outcome is not relevant for the evaluation of the transmitting risk of this subject. Analyses of CNVs in multiple LCRs using ddPCR concealed variations in specific LCRs detected by fiber-FISH. This situation explains the lack of concordance between the data provided by both techniques and reinforces the need to analyse the LCR architecture with LCR-specific methods.

Although the CNVs of pseudogene sequences were not conclusive due the limited sample size, we do believe that the presence of such sequences could be another variable to take into account. Genetic variations in pseudogene sequences located at LCRs have been already proposed as rearrangement promoters^57^. We observed that, in DGS/VCFS parents-of-origin, the presence of a possible *AK129567* pseudogene sequence was less frequent than in the general population. The absence of pseudogene sequences might alter the degree of homology between LCR22-2 and LCR22-4, enhancing their misalignment, which would cause more NAHR events. Nevertheless, as we have just described, the ddPCR technique hampers the assignation of these CNVs to any LCR22. Consequently, we are aware that these pseudogene sequence variations might be located in a LCR that is not involved in the DGS/VCFS reorganization.

Parental inversion polymorphisms have also been proposed as a susceptibility factor for NAHR events in multiple genomic disorders, especially in Williams-Beuren syndrome^20^,^21^,^22^. With regard to the 22q11.2 inversions, in agreement with the work of Gebhardt *et al*.^44^ and Saitta *et al*.^45^, we did not observe any haplotype corresponding to the 3 Mb inversion, either in DGS/VCFS parents-of-origin or in control individuals. These results discard the presence of heterozygous inversions as a predisposing factor for 22q11.2 deletions.

Concerning the role of the *PRDM9* genotype, we did not find differences between the allelic frequencies of controls and DGS/VCFS parent-of-origin populations. Despite the limited sample size, and considering that other authors also failed to identify this association^32^,^46^, the results indicate that there are non-specific *PRDM9* alleles related to DGS/VCFS susceptibility. Accordingly, our data support the idea that the *PRDM9* genotype has a limited influence on NAHR events. Indeed, although the *PRDM9* A allele has been proposed as a predisposing factor for CMT1A or HNPP^33^, this allele is extremely common in Caucasians, suggesting that this genetic feature cannot be a predisposing factor by itself^33^. However, it is interesting to note that the finding of a minor and undescribed allele in DG2F might indicate a possible influence as a NAHR catalyst in this individual. Actually, rare *PRDM9* alleles might contribute to different hotspot patterns that create unusual DSBs at disease-causing 22q11.2 deletion breakpoints.

The risk of transmitting a genomic disorder is a complex trait that could be attributed to a confluence of different genetic characteristics. First, regarding the 22q11.2 genomic architecture, we have raised the possibility that CNVs of segmental duplication blocks within LCR22-2 and LCR22-4 could be considered as a DGS/VCFS predisposing factor. Moreover, our results also suggest a possible implication of pseudogene sequence CNVs. Second, although the *PRDM9* genotype does not seem to be a decisive predisposing factor, we cannot reject its contribution in the definition of the DGS/VCFS transmitting risk due to the possible existence of rare *PRDM9* alleles capable of creating new hot spot patterns. The *PRDM9* genotype and the LCR22 genomic architecture could have a synergistic effect considering that CNVs at LCR22s also vary the number and distribution of recombination motifs.

Our results provided a comprehensive approximation to the analysis of DGS/VCFS predisposing genetic factors in parents-of origin. Although designed as an exploratory study, results suggested that CNVs in LCR22s predispose to sperm 22q11.2 deletions. Nevertheless, further studies is a larger cohort of individuals are needed to confirm the contribution of segmental duplication blocks, and better understand the participation of pseudogenes and rare *PDRM9* alleles to NAHR 22q11.2 susceptibility.

## Methods

### Study population

**Figure 5:**
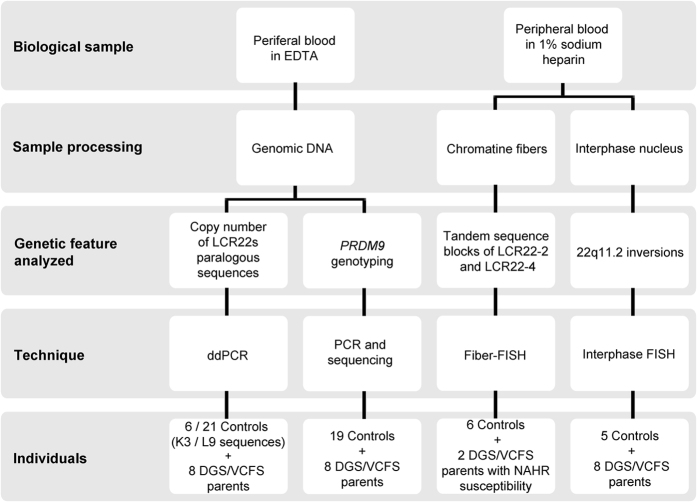
Experimental design.

This study was carried out on six DGS/VCFS fathers-of-origin, two DGS/VCFS mothers-of-origin and a total of 26 control individuals (Fig. 5). To our knowledge, none of them had been exposed to genotoxic agents, and no history of chemotherapy, radiotherapy or chronic illness was recorded. In DGS/VCFS families, the parental origin of the deleted chromosome, and the frequency of sperm 22q11.2 deletions, were previously reported^13^.

All the individuals gave their informed consent in writing to participate in the study and the protocols were approved by the Ethics Commission on Human and Animal Experimentation of the *Universitat Autònoma de Barcelona*. All experiments were conducted in accordance with the informed consent and the approved guidelines.

### Biological samples

Peripheral blood samples were collected in EDTA-containing tubes (5 ml). Genomic DNA was isolated using the Gentra Puregene Blood kit (QUIAGEN Inc.) following the manufacturer’s instructions. Peripheral blood samples were also collected in 1% sodium heparin tubes in eight DGS/VCFS parents-of origin and ten control individuals (10 ml) (Supplementary Table S4 and Fig. 5).

### LCR22-2 and LCR22-4 tandem sequence blocks

#### Slide preparation

Chromatin fibers were obtained according to a previously described protocol^58^. Lymphocytes were isolated from peripheral blood samples in 1% sodium heparin by Ficoll-Paque^TM^ gradient separation. The lymphocyte phase was centrifuged and the pellet was washed in 1x PBS. The lymphocyte pellet was resuspended in 1x PBS to reach a final concentration of 2.10^6^ cells/ml. 10 μl of the lymphocyte isolation was spread and dried on slides. Slides were placed in Shandon Sequenza Coverplates and chromatin fibers were stretched by applying a lysis solution (0.07 M NaOH in ethanol). Fibers were fixed in methanol and stored at −20 °C until they were processed.

#### DNA probe preparation

BAC and fosmid clones were provided by BACPAC Resources Center, CHORI, USA (http://bacpac.chori.org/) and Source BioScience (Supplementary Table S1). We validated probe positioning and efficiency using FISH on metaphase chromosomes (see Methods, 22q11.2 inversions).

FISH probes were purified from BACs and fosmid clones using the QIAprep Spin Miniprep Kit (QIAGEN) and differentially labelled with Spectrum Orange-dUTP (Abbot Molecular) or digoxigenin-11-dUTP (Roche) by Nick Translation (Abbot Molecular). Multiple colour combinations of two delimiting BAC clones and one fosmid were simultaneously hybridized on DNA fiber slides. The proximal BAC was labelled with both Spectrum Orange and digoxigenin-FITC, the fosmid with digoxigenin-FITC and the distal BAC with Spectrum Orange (Fig. 1B).

After the NICK translation reaction, probes were ethanol precipitated with 1 μg/ml salmon testis DNA (GE Healthcare) and 3 M sodium acetate (pH 5.5) at −80 °C for 3 h. Probes were purified by centrifugation at 14,000 rpm for 30 minutes, followed by a wash in 70% ethanol and centrifugation at 14,000 rpm for 6 minutes. The probe pellet was air-dried, resuspended in 1x TE buffer (10 mM Tris, 1 mM EDTA, pH 7.0) at a concentration of 20 ng/μl and stored at −20 °C.

#### FISH on extended chromatin fibers (Fiber-FISH)

Probe combinations were prepared by mixing 400 ng of each labelled DNA probe with 8 μg of Cot1 competitor DNA (Invitrogen) per probe. The probe hybridization mixture was dried on a heating block at 42 °C and the pellet was resuspended in 10 μl of LSI/WCP Hybridization Buffer (Abbot Molecular). Before hybridization, probes were denatured at 75 °C for 5 minutes and pre-annealed at 37 °C for 45 minutes.

Slides were dehydrated in an ethanol series (70, 90 and 100% for 2 minutes each) and dried at room temperature. Chromatin fibers were denatured in 70% formamide/2x SSC at 70 °C for 2 minutes, washed in 2x SSC for 5 minutes and dehydrated in an ethanol series (70, 85 and 100% for 1 minute each). Probes were applied to slides and hybridized in a moist chamber at 37 °C for 48 hours.

Post-hybridization washes were performed in 2x SSC at 42 °C for 3 minutes, followed by 1x PBS at room temperature for 5 minutes. Slides were incubated in a blocking solution (5% dried non-fat milk in 1x PBS/0.05% Tween-20 (Sigma-Aldrich)) at 37 °C for 30 minutes. Then slides were transferred to 1x PBS at room temperature for 5 minutes, followed by one hour incubation at 37 °C in detection solution (1% dried non-fat milk in 1x PBS/0.05% Tween-20 (Sigma-Aldrich) with 1 μg/ml fluorescein-conjugated antidigoxigenin (Roche)). Finally, slides were washed three times in 1x PBS for one minute, dried out at room temperature and counterstained with 4’,6-diamidino-2-phenylindole (DAPI, Vysis).

#### Image acquisition and data analyses

A minimum of 20 informative fibers were scored for every single probe combination on an Olympus BX60 epifluorescence microscope equipped with Isis Imaging System (Metasystems). Fiber-FISH analyses were developed by applying the following assessment criteria adapted from Molina *et al*.^50^: (i) at least three signals of different colour combinations must be observed in a consecutive and linear fashion, ii) two or more signals of the same colour are considered independent when they are separated by a distance twice the distance of every single bead-on-string, and iii) signals are considered informative regardless of the size (Fig. 1D). For each BAC and fosmid combination, we scored the frequency of the fosmid signal numbers that corresponded to the tandem sequence blocks.

### LCR22 paralogous sequences

ddPCR was performed using genomic DNA isolated from peripheral blood samples in EDTA. To achieve an optimal fragmentation of LCR22 tandem sequences, 2 μg of genomic DNA was digested with 20U of *HaeIII* (New England Biolabs) in a final volume of 25 μl containing 1x NEB4 buffer for 1 h at 37 °C. The digest was diluted to a final concentration of 6 ng/μl and 9 ng were assayed per 20 μl ddPCR reaction. Primers and probes were designed for L9 and K3 fosmid clones (reported abnormal CNVs of tandem sequence blocks in DGS/VCFS fathers-of-origin). L9 fosmid sequences were assayed using forward primer CAGTGGGACTCTCATCAAAC, reverse primer AGGAGCGAGAAATAGAGTCC and probe FAM-AAAGTTGCCATCAGCCAGATGCCAG-Iowa Black^®^FQ. The 116 bp amplicon corresponded to *AK129567* gene. K3 fosmid sequences were assayed using forward primer ATTTTGCGGTGTCAGAAGGT, reverse primer CCTCTCAGTGACTGTCTCCT and probe FAM-CGGGAGGCAAGCAGTCCCTGGTCC-Iowa Black^®^FQ. The 92 bp amplicon corresponded to *AK302545* gene. Both assays were duplexed with an EIF2C1 reference assay probe labelled with HEX/Iowa Black^®^FQ (PrimePCR™ ddPCR™ Copy Number Assay EIF2C1, Bio-Rad). Amplification conditions were 95 °C for 10 min (1 cycle), 94 °C for 30 s and 55.5 °C × 60 s (40 cycles), 98 °C × 10 min (1 cycle), and 4 °C hold. Each assay was performed in two replicate ddPCR wells. After PCR, the 96-well PCR plate was loaded on the droplet reader (Bio-Rad). Positive and negative droplet counts were analysed combining data from both replicates and using QuantaSoft analysis software (Bio-Rad). For each replicate, we obtained an average number of the amplified copies from two haplotypes.

### 22q11.2 inversions

#### Slide preparation

Lymphocytes from peripheral blood samples (collected in 1% sodium heparin) were cultured in phytohaemagglutinin-stimulated medium at 37 °C in a 5% CO_2_ atmosphere for 72 h. Cells were treated with colcemid, swelled after a hypotonic treatment (75 mM KCl) and fixed with 3:1 methanol-acetic acid.

#### DNA probe preparation

A customized combination of BAC clones RP11-66F9 (abbr. F9), RP11-505B16 (abbr. B16) and RP11-47L18 (abbr. L18) was used. Probes were labelled with Aqua-dUTPs (Abbott Molecular), Spectrum Green-dUTPs (Abbott Molecular) and Spectrum Orange-dUTPs (Abbott Molecular), respectively, as described previously for the fiber-FISH assays.

Probe combinations were prepared by mixing 200 ng of each labelled DNA probe with 4 μg of Cot1 competitor DNA (Invitrogen) per probe. The probe hybridization mixture was dried on a heating block at 42 °C and the pellet was resuspended in 5 μl of LSI/WCP Hybridization Buffer (Abbot Molecular).

#### Interphase FISH

Slides were washed twice in 2x SSC for 3 minutes, dehydrated in an ethanol series (70, 90 and 100% for 2 minutes each) and dried at room temperature. Slides were denatured in 70% formamide/2x SSC at 73 °C for 2 minutes, and dehydrated in an ethanol series (70, 85 and 100% for 1 minute each). Probes were applied to slides and hybridized in a moist chamber at 37 °C for 24 hours. Post-hybridization washes were performed in 1x SSC with 0.3% NP-40 at 73 °C and in 2x SSC with 0.1% NP-40 at room temperature for one minute in each solution.

#### Image acquisition and data analyses

Analyses were performed using an Olympus BX60 epifluorescence microscope equipped with an Isis Imaging System (Metasystems). Only those signal combinations with a clear distribution of two clustered signals (in close proximity or overlapping) and one separated signal were considered informative. The distant signal must be separated from the two others by at least a two-fold longer distance compared with the separation of the clustered signals. This probe configuration allowed us to distinguish the normal haplotype (N) and the 3 Mb inversion haplotype (inv) (Fig. 1C and E). A minimum of 100 informative signal combinations were analysed per sample. Metaphase chromosomes were also observed to test probe specificity.

### *PRDM9* genotype

The *PRDM9* zinc finger array was amplified by PCR in genomic DNA isolated from peripheral blood samples in EDTA. PCR was performed using PN0.6 F and PN2.5 R primers (PN0.6 F: TGAGGTTACCTAGTCTGGCA, PN2.5 R: ATAAGGGGTCAGCAGACTTC)^33^, 3% DMSO and BioTaq (Bioline). Amplifications were performed as follows: 95 °C for 3 min; 45 cycles of 95 °C for 30 s, 62 °C for 30 s and 72 °C for 105 s. PCR products were purified with PCR DNA purification kit (Thermo Scientific) and subjected to bidirectional Sanger sequencing with the primers PN1.2 F and PN2.4 R (PN1.2 F: TGAATCCAGGGAACACAGGC and PN2.4 R: GCAAGTGTGTGGTGACCACA)^33^ using BigDye Terminator v3.1 Cycle Sequencing (Applied Biosystems) on a ABI 3130XL Genetic Analyzer (Applied Biosystems).

In order to determine which *PRDM9* alleles presented each subject, the zinc finger array sequences were aligned and compared to published data^31^,^33^,^46^,^59^.

### Statistical analysis

Data were statistically analysed using Graph Pad Prism 5.03. The statistical significance of the results was determined by a *p-value* less than 0.05.

Data from ddPCR replicates were averaged. To assess CNVs between LCR22 paralogous sequences from controls and DGS/VCFS fathers-of-origin, the mean copy number of each fosmid was compared between both populations using a Mann-Whitney test. In order to determine whether fiber-FISH and ddPCR technics were equivalent, the sum of the fosmid blocks of LCR22-2 and LCR22-4 per individual and the total copy number of LCR22 paralogous sequences were compared with a Spearman correlation test.

In the 22q11.2 inversion analyses, differences between haplotype frequencies from control and DGS/VCFS parent-of-origin populations were analysed by applying a Mann-Whitney test.

To ascertain the relevance of *PRDM9* alleles in the risk for DGS/VCFS, the frequency of each allele was compared between controls and DGS/VCFS parents-of-origin with a Fisher exact test.

## Additional Information

**How to cite this article**: Vergés, L. *et al*. An exploratory study of predisposing genetic factors for DiGeorge/velocardiofacial syndrome. *Sci. Rep.* **7**, 40031; doi: 10.1038/srep40031 (2017).

**Publisher's note:** Springer Nature remains neutral with regard to jurisdictional claims in published maps and institutional affiliations.

## Supplementary Material

Supplementary Tables

## Figures and Tables

**Table 1:** CNVs of tandem sequence blocks.

Sample ID	LCR22.2				LCR22.4					
	K3	L9	B22	L21	K3	L9	B22	M12	I18	
Controls										
C1	5\7	5\6	2\3	1\2	2\3	2\2	1\2	2\2	2\2	
C2	7\9	5\10*	3\5*	1\2	2\2	2\2	1\2	2\2	2\3	
C3	3\5	3\5	1\2	1\2	1\2	1\2	1\1	2\2	2\2	
C4	3\5	3\5	2\3	1\1	2\2	2\2	1\1	2\2	2\2	
C5	3\3	2\5	2\4	1\2	1\2	1\2	2\2	2\2	2\2	
C6	3\5	2\4	1\2	1\1	1\2	2\2	1\2	2\2	2\2	
Normal range**	3–9	2–6	1–4	1–2	1–3	1–2	1–2	2–2	2–3	
DGS/VCFS fathers***										
DG2F	**2\2**	3\6	2\3	1\2	2\2	2\**3**	1\2	2\2	2\2	
DG5F	5\8	**8\8**	2\3	1\2	2\2	2\**3**	1\2	2\2	2\2	

^*^Outlier. ^**^Ranges established with the lowest and the highest values in control individuals excluding outliers. ^***^DGS/VCFS fathers-of-origin with increased number of 22q11.2 sperm deletions (Vergés *et al*.^13^). The two haplotypes for each individual are separated by a backslash. Bold numbers indicate CNVs out of the normal range.

**Table 2:** Genetic features assessed in DGS/VCFS parents-of-origin.

Sample ID	Relationship	Deletion and duplication frequency in sperm			Segmental duplication blocks		Pseudogene sequences	22q11.2 inversion	*PRDM9* genotype
		22q11.2^1^	15q11-q13^2^	7q11.2^2^	LCR22-2	LCR22-4			
DG1F	DGS/VCFS father	Normal	Normal	Normal	—	—	5	Absence	A/A
DG2F	DGS/VCFS father	Increased	Normal	Normal	Decreased	Increased	0	Absence	A/L50
DG3F	DGS/VCFS father	Normal	Normal	Normal	—	—	0	Absence	A/L9
DG5F	DGS/VCFS father	Increased	Normal	Normal	Increased	Increased	3	Absence	A/A
DG6M	DGS/VCFS mother	—	—	—	—	—	2	Absence	A/A
DG7F	DGS/VCFS father	Normal	Normal	Normal	—	—	0	Absence	A/A
DG8F	DGS/VCFS father	Normal	Normal	Normal	—	—	0	Absence	A/L24
DG9M	DGS/VCFS mother	—	—	—	—	—	0	Absence	A/A

^1^Data extracted from Vergés *et al*.^13^.

^2^Unpublished data.
