# Supplementary material for: An exploratory study of predisposing genetic factors for DiGeorge/velocardiofacial syndrome
Source: Sci Rep. 2017 Jan 6;7:40031. doi: 10.1038/srep40031 (PMC5216377; doi:10.1038/srep40031)
Supplement: Supplementary Tables [file srep40031-s1.pdf]

An exploratory study of predisposing genetic factors for DiGeorge/velocardiofacial syndrome

Laia Vergés, Francesca Vidal, Esther Geán, Alexandra Alemany-Schmidt, Maria Oliver-Bonet and Joan Blanco

SUPPLEMENTARY TABLES

Supplementary Table S1. Characteristics of the BAC and fosmid clones (UCSC Assembly Feb 2009).

	Clone	Abbreviation	UCSC reference	Size (Kb)	UCSC genes
BAC clones	RP11-66F9[1]	F9	RP11-66F9	175.3	*GAP4*, *CECR7*
	RP11-163A10[2]	A10	RP11-163A10	183.1	*HIRA*
	RP11-505B16[1]	B16	RP11-505B16	171.3	*PI4KA*
	RP11-47L18[1]	L18	RP11-47L18	186.6	*UBE2L3*, *PPIL2*
Fosmid clones	WI2-938L9[1]	L9	G248P80545F5	41.6	*USP18*, *AK129567*
	WI2-451K3[1]	K3	G248P8704F2	44.3	*AK302545*, *GGT3P*
	WI2-1268B22[1]	B22	G248P82259A11	41.9	*GGT3P*
	WI2-1822L21[1]	L21	G248P86641F11	39.0	*DGCR6*, *PRODH*
	WI2-2886M12[1]	M12	G248P88386G6	44.2	*RIMBP3C*
	WI2-3344I18[1]	I18	G248P800126E9	45.5	*PI4KAP2*

[1] BACPAC Resources Center, CHORI
[2] Source BioScience

Supplementary Table S2. 22q11.2 inversion results in controls and DGS/VCFS transmitting parents.

Case	Total nuclei	Informative haplotypes (%)	Haplotypes N (%)	Haplotypes Inv (%)	Haplotypes Other (%)*	Genotype
Controls						
C3	87	102 (58.62)	98 (96.08)	1 (0.98)	3 (2.94)	N/N
C7	69	101 (73.19)	96 (95.05)	2 (1.98)	3 (2.97)	N/N
C9	77	102 (66.23)	96 (94.12)	3 (2.94)	3 (2.94)	N/N
C11	77	104 (67.53)	101 (97.12)	1 (0.96)	2 (1.92)	N/N
C14	75	103 (68.67)	101 (98.06)	1 (0.97)	1 (0.97)	N/N
% ±SEM		66.85 ±2.37	96.08 ±0.70	1.57 ±0.40	2.35 ±0.40	
DGS/VCFS transmitting parents						
DG1F	77	103 (66.88)	100 (97.09)	3 (2.91)	0	N/N
DG2F	112	119 (53.13)	116 (97.48)	0	3 (2.52)	N/N
DG3F	86	102 (59.30)	101 (99.02)	1 (0.98)	0	N/N
DG5F	103	110 (53.40)	104 (94.55)	3 (2.73)	3 (2.73)	N/N
DG6M	78	103 (66.03)	101 (98.06)	2 (1.94)	0	N/N
DG7F	93	102 (54.84)	101 (99.02)	0	1 (0.98)	N/N
DG8F	93	102 (54.84)	97 (95.10)	2 (1.96)	3 (2.94)	N/N
DG9M	80	101 (63.13)	96 (95.05)	2 (1.98)	3 (2.97)	N/N
% ± SEM		57.96 ±2.02	96.76 ±0.64	1.51 ±0.40	1.73 ±0.50	

*Hybridization errors and non-expected associations.

Supplementary Table S3. *PRDM9* genotyping of control individuals and DGS/VCFS transmitting parents.

	Sample ID	Zinc finger array haplotype 1	Zinc finger array haplotype 2	Alleles
Control individuals	C7	ABCDDECFGHFIJ	ABCDDECFGHFIJ	A/A
	C8	ABCDDECFGHFIJ	ABCDDECFGHFIJ	A/A
	C9	ABCDDECFGHFIJ	ABCDDECFGHFIJ	A/A
	C10	ABCDDECFGHFIJ	ABCDDECFGHFIJ	A/A
	C11	ABCDDECFGHFIJ	ABCDDECFGHFIJ	A/A
	C12	ABCDDECFGHFIJ	ABCDDECFGHFIJ	A/A
	C13	ABCDDECFGHFIJ	ABCDDECFGPFQJ	A/L9
	C14	ABCDDECFGHFIJ	ABCDDECFGHFIJ	A/A
	C15	ABCDDECFGHFIJ	ABCDDECFGHFIJ	A/A
	C16	ABCDDECFGHFIJ	ABCDDECFGHFIJ	A/A
	C17	ABCDDECFGHFIJ	ABCDDECFGHFIJ	A/A
	C19	ABCDDECFGHFIJ	ABCDDECFGHFQJ	A/L26
	C20	ABCDDECFGHFIJ	ABCDDECFGHFIJ	A/A
	C21	ABCDDECFGHFIJ	ABCDDECFGKFQJ	A/L20
	C22	ABCDDECFGHFIJ	ABCDDECFGHFIJ	A/A
	C23	ABCDDECFGHFIJ	ABCDDECFGHFQJ	A/L26
	C24	ABCDDECFGHFIJ	ABCDDCCFGHFIJ	A/B
	C25	ABCDDECFGHFIJ	ABCDDECFGHFIJ	A/A
	C26	ABCDDECFGHFIJ	ABCDDECFGHFIJ	A/A
DGS/VFCS transmitting parents	DG1F	ABCDDECFGHFIJ	ABCDDECFGHFIJ	A/A
	DG2F	ABCDDECFGHFIJ	ABCCCCC?FGHFIJ*	A/L50
	DG3F	ABCDDECFGHFIJ	ABCDDECFGPFQJ	A/L9
	DG5F	ABCDDECFGHFIJ	ABCDDECFGHFIJ	A/A
	DG6M	ABCDDECFGHFIJ	ABCDDECFGHFIJ	A/A
	DG7F	ABCDDECFGHFIJ	ABCDDECFGHFIJ	A/A
	DG8F	ABCDDECFGHFIJ	ABCDDECFTPFQJ	A/L24
	DG9M	ABCDDECFGHFIJ	ABCDDECFGHFIJ	A/A

* Technical difficulties hamper the accurately sequencing of all the zinc finger repeats allele.

Supplementary Table S4. Biological samples collected from DGS/VCFS transmitting parents.

Sample ID	Relationship	Sperm sample[1]	Peripheral blood sample		
			Genomic DNA[1]	Chromatin fibers	Interphase nucleus
DG1F	DGS/VCFS father	Yes	Yes	No	Yes
DG2F	DGS/VCFS father	Yes	Yes	Yes	Yes
DG3F	DGS/VCFS father	Yes	Yes	No	Yes
DG5F	DGS/VCFS father	Yes	Yes	Yes	Yes
DG6M	DGS/VCFS mother	-	Yes	No	Yes
DG7F	DGS/VCFS father	Yes	Yes	No	Yes
DG8F	DGS/VCFS father	Yes	Yes	No	Yes
DG9M	DGS/VCFS mother	-	Yes	No	Yes

[1]Samples processed in Vergés et al. (2014)
